# Temporal and spatial trends of ischemic heart disease burden in Chinese and subgroup populations from 1990 to 2016: socio-economical data from the 2016 global burden of disease study

**DOI:** 10.1186/s12872-020-01530-0

**Published:** 2020-05-24

**Authors:** Chenran Wang, Chunping Wang, Mi Liu, Zhe Chen, Shiwei Liu

**Affiliations:** 1 “Health Shandong” Major Social Risk Prediction and Governance Collaborative Innovation Center, School of Public Health and Management, Weifang Medical University, No.7166 Baotong Xi Street, Weicheng District, Weifang, 261053 China; 2 Chinese Center for Disease Control and Prevention, No.27 Nanwei Road, Xicheng District, Beijing, 100050 China

**Keywords:** China, Burden of disease, Disability-adjusted life years, Ischemic heart disease, Temporal and spatial trend

## Abstract

**Background:**

Ischemic heart disease (IHD) is the leading cause of premature death which poses public health challenges worldwide. Previous studies focused on the overall population in China. However, variations in temporal and spatial patterns across subgroups remain unknown. This study was to analyze how the IHD burden among Chinese and subgroup populations changes in response to temporal and spatial trends from 1990 to 2016.

**Methods:**

Based on data from the updated estimate in the 2016 Global Burden of Disease (GBD) study, we used years lived with disability (YLDs), years of life lost (YLLs), and disability-adjusted life years (DALYs) to describe the IHD burden. The percentage and annual average percentage changes were applied to illustrate temporal and spatial variations of the IHD burden stratified by age, sex, and province, over the periods 1990–2016, 1990–2005, and 2005–2016. We estimate population-attributable fraction (PAF) for 24 modifiable risk factors at the provincial level in 2016.

**Results:**

YLD rates, YLL rates, and DALY rates for IHD underwent a notable increase among all age groups and increased by 119.4, 83.3, and 84.5% nationally from 1990 to 2016. In YLD rates, a greater increase was seen in females (124.4%) compared to males (114.0%), while males experienced a more substantial increase than that in females in YLL rates (99.3% vs. 60.5%) and DALY rates (99.7% vs. 63.2%) from 1990 to 2016. Compared with 1990–2005, annual average changes in the overall population in YLL rates (3.5% vs. 1.8%) and DALY rates (3.5% vs. 1.9%) showed a tardier increase whereas an opposite increasing trend of YLD rates (3.5% vs. 4.0%) was observed between 2005 and 2016. Geographically, all provinces saw declines in the YLLs/YLDs ratio from 2005 to 2016, with seventeen of thirty-three provinces showing an upward trend between 1990 and 2005. Most provinces witnessed a remarkable upsurge in the age-standardised DALY rate from 1990 to 2016 whereas the economically advantaged region Macao (52.2%) saw the most marked reduction. High systolic blood pressure and high LDL cholesterol remained the two leading risk factors of IHD in all provinces in 2016. Diet high in sodium was the leading behavioral risks in twenty-eight provinces with smoking heading the list in five provinces.

**Conclusions:**

China has made significant achievements in preventing premature death from IHD along with the increased risk of disability. Substantial disparities in temporal and spatial trends of the IHD burden emphasize concerns for elderly men and those in economically disadvantaged regions with resource constraints. Regional differences in the IHD burden can be partly explained by modifiable risk factors. By having identified these disparities, targeted IHD prevention and control strategies will help to bridge these gaps.

## Background

Ischemic heart disease (IHD), the main subcategory of cardiovascular diseases (CVDs), has been identified as the main chronic non-communicable disease (NCDs). The World Health Organization (WHO) projected that by 2020, IHD will become the primary cause of global death and disability [1]. As the chief contributor to the increase of global CVD burden, IHD caused approximately 26.2 million morbidity and 9.5 million mortality worldwide in 2016 [2, 3]. In China, the world’s largest developing country with > 1.4 billion population, the improved medical care and living standards have raised healthy life expectancy (HALE) from 59.8 years in 1990 to 69.9 years in 2016 [4]. However, the disease burden from CVDs has become a serious public health problem with IHD being the underlying cause of these growing health concerns [5, 6]. The increased IHD mortality rate was responsible for the rise in the overall CVDs death and IHD is projected to be the leading cause of death in China [7].

Over the past two decades, demographic shifts, transitions in life styles, and enhanced health care have had a widespread and far-reaching influence on epidemiology of IHD [8]. The temporal and spatial trends of IHD burden should therefore be major concerns; however, previous studies paid disproportionately much attention to the overall population, with variations across subgroup populations in China remaining unknown. More importantly, these studies were of inadequate perspectives with limited time frames, lacking specific and detailed trend analyses of annual average transition, and failing to provide comprehensive temporal and spatial assessments which considered both demographic characteristics and provincial disparities [9–11]. To bridge these persistent gaps significant to informing public health, this study focuses on the systematic evaluation of variations in age-, sex-, and province-specific IHD burdens during1990–2016, based on data from the 2016 Global Burden of Disease (GBD) study. Targeted preventive strategies and initiatives are then proposed to mitigate the IHD burden and allocate health resources effectively [12].

## Methods

### GBD 2016 synopsis

The GBD study was a statistical report examining the health status of the global population. It provided comprehensive, dynamic, and accurate epidemiological models to estimate the burden of disease, injuries, and risk factors, which would help to inform governments of health policies to prioritize resource allocation and meet public health needs at global, regional, national, and sub-national levels.

The 2016 GBD study provided years lived with disability (YLDs) of 328 diseases or injuries, disability-adjusted life years (DALYs) of 333 diseases or injuries, 84 risk factors, and the disease burden of 2892 sequelae in 195 countries and regions around the world from 1990 to 2016 [2–5]. Chinese sub-national assessments adopting locational specific methodology had been put in place since the 2013 GBD study, based on cooperation between the Chinese Center for Disease Control and Prevention (CDC) and the Institute for Health Metrics and Evaluation (IHME) at the University of Washington in the US [13]. The detailed of the study have been fully described elsewhere [2–5].

### Data sources

Updated data on the cause of death were mainly obtained from the Disease Surveillance Points System, the Maternal and Child Surveillance System, and the Death Cause Reporting System of the China CDC. The incidence and prevalence data were collected from disease surveillance, relevant literature researches, and national investigations. New systematic reviews and high quality meta-analyses associated with nationwide scientific researches, including the Fifth National Health Service Survey and the Chronic Disease and Risk Factor Surveillance, were also introduced into the GBD 2016 study [14, 15].

The GBD study collected globally accessible data and used unified methods to ensure the results were comparable, and nationally and regionally representative. We also conformed to the Guidelines for Accurate and Transparent Health Estimates Reporting (GATHER) to ensure the transparency and reproducibility of results. IHD was defined as per the International Statistical Classification of Diseases (10th Revision) (*ICD-10*) with diagnosis codes I20-I25 [3]. No permissions were required to use the data of this study.

### Estimation of disease burden

The Bayesian meta-regression model DisMod-MR 2.1 was used as the main model to estimate the prevalence of non-fatal diseases. The Cause of Death Ensemble Model (CoDEM) was adopted to appraise cause-specific mortality [4]. The indicators describing the IHD burden in this study included YLDs, years of life lost (YLLs), and DALYs. YLDs were calculated based on disability weights from previous population-surveys in GBD 2013 and GBD 2015 studies [2, 4], and YLLs were calculated by multiplying life expectancy by cause-specific deaths. DALYs were the sum of YLDs and YLLs. The methodology in detail for the above metrics has been published previously [2–4]. The age-standardised rate was standardized by the GBD 2016 global population. We applied percentage and annual average percentage changes to demonstrate variations of the IHD burden among Chinese and subgroup populations across all thirty-three provinces/regions (thirty-one mainland provinces, Hong Kong Special Administrative Region, and Macao Special Administrative Region) of China over the years 1990–2016 including the periods 1990–2005 and 2005–2016.

### Risk factors

Comparative risk assessment (CRA) framework was used to estimate the excess IHD DALYs attributable to behavioral, metabolic, and environmental risk factors. We used population-attributable fraction (PAF) to estimate the avoidable proportion if the exposure to a specific risk could be reduced to theoretical minimum risk exposure level (TMREL). The methodology in detail for risk assessments has been published elsewhere [5]. 24 risk-IHD pairs in causal relationship at the provincial level in 2016 were established in this study.

## Results

### IHD burden trends by age and sex

Table 1 shows the percentage change of YLD, YLL, and DALY rates for IHD in age- and sex-specific populations. From 1990 to 2016, YLD, YLL, and DALY rates in the overall population denoted an increase of 119.4, 83.3, and 84.5%, respectively. A marked discrepancy in the variation of the IHD burden between sexes was observed: a greater increase in the YLD rate was seen in females (124.4%) compared to males (114.0%), while males experienced a more rapid increase in YLL rates (99.3% vs. 60.5%) and DALY rates (99.7% vs. 63.2%). The most rapid increase in the DALY (35.1%) and YLL rates (34.9%) for IHD were seen in population aged 70+ years from 1990 to 2016.

**Table 1:** Variation in burden of IHD stratified by age and sex with percentage change from 1990 to 2016, 1990 to 2005, and 2005 to 2016 in China

		Male			Female			Total		
		% change	% annual average change		% change	% annual average change		% change	% annual average change	
	age group	1990–2016	1990–2005	2005–2016	1990–2016	1990–2005	2005–2016	1990–2016	1990–2005	2005–2016
**YLD rate**	< 5 y	16.3	0.6	0.7	20.3	0.8	0.6	18.2	0.7	0.7
	5 **~** 14 y	18.3	0.9	0.5	23.0	1.0	0.6	20.5	1.0	0.5
	15 **~** 49 y	62.1	2.3	1.8	78.9	2.9	2.2	70.5	2.6	2.0
	50 **~** 69 y	25.3	0.8	1.1	27.4	0.7	1.4	26.6	0.7	1.3
	70+ y	38.7	1.3	1.4	42.7	1.1	2.0	40.5	1.2	1.7
	total	114.0	3.5	3.7	124.4	3.5	4.3	119.4	3.5	4.0
**YLL rate**	< 5 y	− 85.5	− 3.7	− 6.1	− 86.3	−4.0	−6.0	−85.9	− 3.9	−6.0
	5 **~** 14 y	− 68.1	−3.0	− 3.9	− 67.5	−3.1	− 3.6	− 67.7	− 3.0	− 3.8
	15 **~** 49 y	36.6	1.9	0.6	−19.6	0.0	−1.8	16.8	1.2	−0.1
	50 **~** 69 y	11.9	0.8	0.1	−21.9	0.0	−2.0	− 2.1	0.4	− 0.7
	70+ y	39.6	2.1	0.6	27.9	2.4	−0.6	34.9	2.3	0.0
	total	99.3	3.7	2.6	60.5	3.2	0.8	83.3	3.5	1.8
**DALY rate**	< 5 y	−83.1	−3.6	−5.7	−84.2	−3.9	−5.6	−83.6	−3.8	−5.7
	5 **~** 14 y	−60.3	−2.6	−3.2	− 57.0	−2.6	− 2.7	− 58.8	−2.6	− 2.9
	15 **~** 49 y	37.3	1.9	0.6	−14.7	0.2	−1.5	18.7	1.3	0.0
	50 **~** 69 y	12.3	0.8	0.1	−19.7	0.0	−1.8	−1.2	0.4	−0.7
	70+ y	39.6	2.1	0.6	28.4	2.4	−0.5	35.1	2.3	0.1
	total	99.7	3.7	2.6	63.2	3.2	0.9	84.5	3.5	1.9

Annual average percentage changes in the IHD burden are shown in Table 1. Compared with 1990–2005, the YLL (1.8% vs. 3.5%) and DALY rates (1.9% vs. 3.5%) experienced slower increases between 2005 and 2016, whereas YLD rates (4.0% vs. 3.5%) showed an opposite increasing trend. For subgroup populations, there was an evident reduction in the annual average percentage change in YLL and DALY rates among population aged < 70 years during 2005–2016, but an increase was observed in the YLD rate among all age groups, especially those aged 50 ~ 69 and above 70 years. No difference in YLL rates in the ≧70-year-old age group was observed over the same period.

**Fig. 1:**
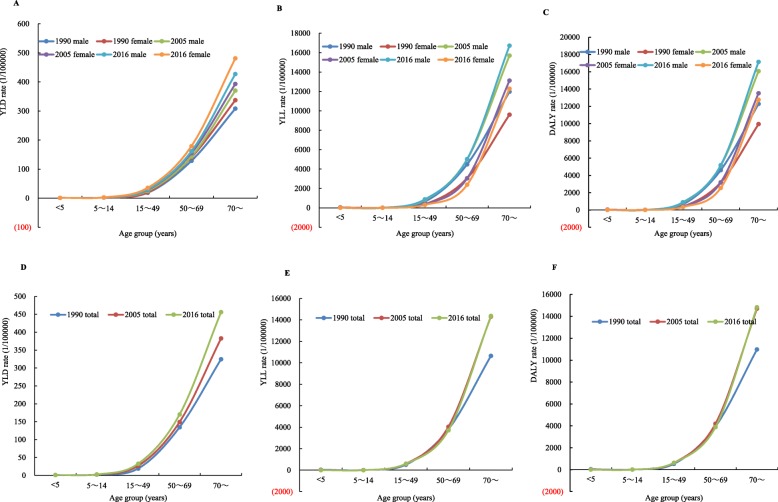
Transition of disease burden caused by IHD by age-sex-year in China. **a** YLD rate of IHD stratified by age and sex in China in 1990, 2005, and 2016 **b** YLL rate of IHD stratified by age and sex in China in 1990, 2005, and 2016 **c** DALY rate of IHD stratified by age and sex in China in 1990, 2005, and 2016 **d** YLD rate of IHD in both sexes combined in China in 1990, 2005, and 2016 **e** YLL rate of IHD in both sexes combined in China in 1990, 2005, and 2016 **f** DALY rate of IHD in both sexes combined in China in 1990, 2005, and 2016

The longitudinal age curves of the YLD, YLL, and DALY rates by sex and year are shown in Fig. 1 (a-f). For males, females, and both sexes combined, YLD, YLL, and DALY rates increased from ages < 5 and reached at peak at 70+ years old in 1990, 2005, and 2016. YLL and DALY rates for IHD in males were higher than those in females who (except for those aged 5 ~ 14 years) had higher YLD rates.

### IHD burden trends by province

#### Spatial variation in YLD and YLL

Despite higher YLLs than YLDs being consistently observed in all regions of China, a substantial decline (16.5%) in the YLLs/YLDs ratio for both sexes was observed from 28.9 in 1990 to 24.1 in 2016. The YLLs/YLDs ratio varied across provinces. While Guizhou (6.4%), Yunnan (6.3%), and Hunan (4.9%) saw an increase in the YLLs/YLDs ratio, the remaining thirty provinces showed a decrease from 1990 to 2016. While sixteen provinces saw an increase and seventeen saw a decrease in the YLLs/YLDs ratio from 1990 to 2005, all provinces in China saw a reduction afterwards (Table 2).

**Table 2:** YLLs/YLDs and age-standardised DALY rates from IHD with percentage change from 1990 to 2016, in provinces of China

	YLLs/YLDs				Age-standardised DALY rate per 100,000people			
Provinces	1990	2005	2016	%change	1990	2005	2016	%change
China	28.9	28.9	24.2	−16.5	1818.1	2150.8	2002.1	10.1
Anhui	23.1	24.8	22.6	−2.3	1502.5	1840.6	1824.9	21.5
Beijing	24.9	23.3	15.6	−37.3	2020.3	2129.1	1612.9	−20.2
Chongqing	22.1	21.6	18.5	−16.3	1355.2	1515.4	1365.1	0.7
Fujian	20.3	21.0	14.4	−29.2	1285.4	1478.7	1138.5	−11.4
Gansu	30.5	33.6	28.0	−8.1	1741.2	2322.6	2206.2	26.7
Guangdong	24.1	23.8	19.0	−21.2	1734.7	1927.1	1697.4	−2.2
Guangxi	29.6	33.8	28.5	−3.6	1791.4	2298.1	2132.9	19.1
Guizhou	25.6	28.0	27.2	6.4	1505.2	1910.7	2009.1	33.5
Hainan	31.3	27.5	25.0	−20.0	1963.7	1862.3	1899.9	−3.3
Hebei	36.2	36.6	30.9	−14.7	2292.4	2926.7	2766.3	20.7
Heilongjiang	46.7	39.9	21.0	−55.1	3268.3	3518.9	3336.9	2.1
Henan	37.1	38.1	30.2	−18.6	2376.2	3035.3	2742.6	15.4
Hubei	25.6	26.5	22.9	−10.7	1696.0	1999.1	1805.2	6.4
Hunan	27.8	30.3	29.1	4.9	1704.7	2260.1	2337.6	37.1
Inner Mongolia	41.5	39.0	29.7	−28.3	2981.9	3594.9	2998.8	0.6
Jiangsu	16.5	16.7	12.1	−26.7	1006.9	1163.5	978.4	−2.8
Jiangxi	29.0	27.0	23.5	−18.9	1747.9	1886.3	1729.4	−1.1
Jilin	53.5	45.9	36.1	−32.6	3408.1	3646.5	3158.6	−7.3
Liaoning	31.0	28.0	23.2	−25.2	2316.1	2539.6	2468.0	6.6
Ningxia	36.2	35.9	30.1	−16.7	2428.6	3006.8	2849.2	17.3
Qinghai	37.6	38.1	34.7	−7.9	2147.7	2632.7	2784.6	29.7
Shaanxi	40.8	37.4	33.3	−18.4	2187.3	2425.9	2536.0	15.9
Shandong	30.8	30.4	25.6	−16.8	1914.5	2372.2	2231.4	16.6
Shanghai	10.8	10.2	8.6	−20.3	789.8	800.8	742.7	−6.0
Shanxi	33.2	29.9	27.7	−16.6	2236.2	2410.9	2446.6	9.4
Sichuan	24.1	24.9	20.0	−17.1	1430.5	1659.3	1498.5	4.8
Tianjin	28.3	28.9	22.4	−20.8	2332.3	2918.2	2410.3	3.3
Tibet	47.7	58.0	42.4	−11.0	2416.3	3497.3	2852.1	18.0
Xinjiang	51.9	59.7	46.9	−9.6	3179.9	4127.1	3632.7	14.2
Yunnan	29.3	31.5	31.1	6.3	1525.9	1879.3	2044.6	34.0
Zhejiang	16.5	14.1	10.9	−34.1	963.4	922.6	790.6	−17.9
Hong Kong Special Administrative Region of China					1370.8		743.6	
	18.6	11.3	8.9	−51.8		919.9		−45.8
Macao Special Administrative Region of China					2758.1		1317.2	
	29.2	18.5	12.3	−57.8		1813.5		−52.2

**Fig. 2:**
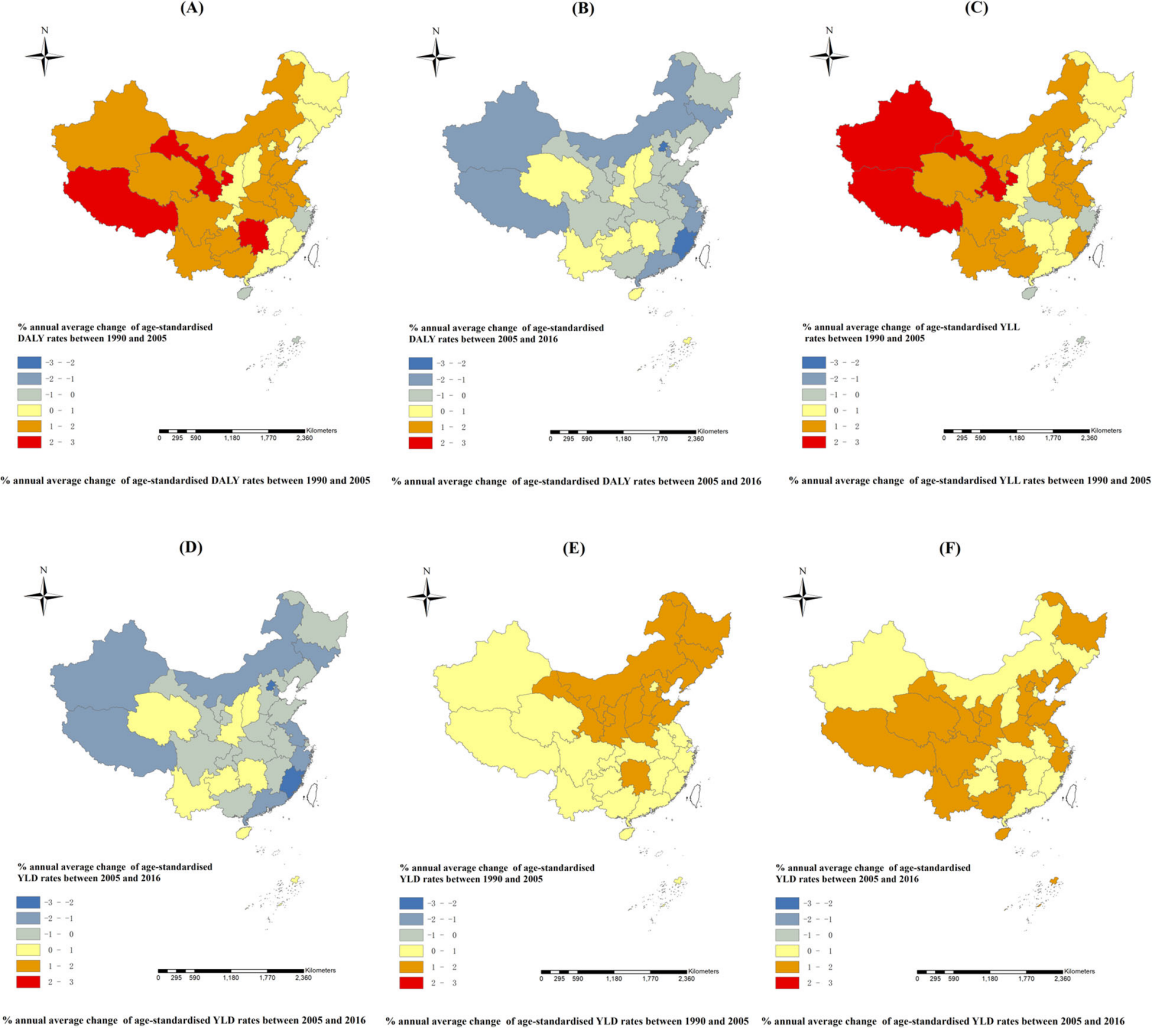
Annual average changes of age-standardised DALY, YLL, and YLD rates for IHD in China from 1990 to 2005, 2005–2016

Comparison maps of age-standardised YLD and YLL rates across provinces were employed to visualize the variation in IHD burden before and after 2005 (Fig. 2, c-f). Age-standardised YLD rates in all provinces increased both in 1990–2005 and 2005–2016, with a larger increase seen in seventeen provinces and slower increases in sixteen provinces from 2005 to 2016. The age-standardised YLL rates in most provinces increased from 1990 to 2005 except for several economically advantaged regions such as Hainan, Zhejiang, Hong Kong, and Macro. Twenty-six provinces saw the reduction in age-standardised YLL rates between 2005 and 2016.

#### Spatial variation in DALY

The age-standardised DALY rate increased by 10.1% from 1818.1 per 100,000 in 1990 to 2002.1 per 100,000 in 2016. Geographic variation in the age-standardised DALY rate from 1990 to 2016 between provinces was observed. Most provinces in China showed a marked increase in age-standardised DALY rates since 1990, whereas certain economically developed provinces had an opposite descending trend. For example, a significant decrease in age-standardised DALY rates between 1990 and 2016 was particularly notable in Macao (52.2%), Hong Kong (45.8%), and Beijing (20.2%), whereas the most substantial increase was observed in Hunan (37.1%) followed by Yunnan (33.9%) and Guizhou (33.5%).

The annual percentage change of age-standardised DALY rates varied across provinces of China. Twenty-nine provinces showed an increase in age-standardised DALY rates with the remaining four showing a decrease from 1990 to 2005, while the age-standardised DALY rates increased in seven provinces and decreased in the remaining twenty six from 2005 to 2016. In some southeastern coastal provinces, such as Fujian, Guangdong, and Zhejiang, a slower annual increase was observed from 1990 to 2005 but a decrease was seen after 2005 (Fig. 2, A-B).

### Province-level modifiable risk factors

**Fig. 3:**
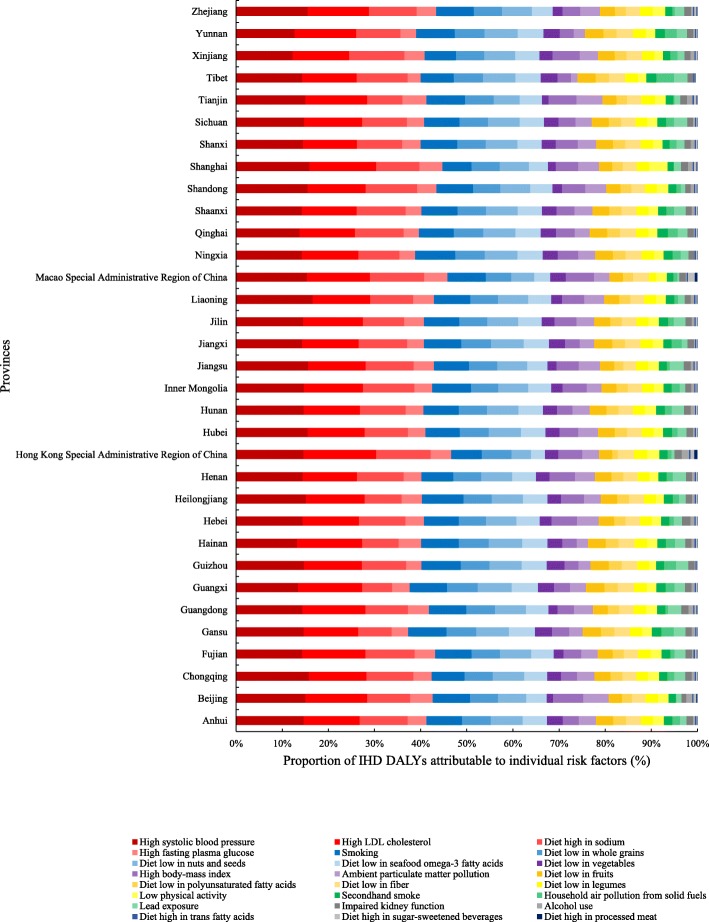
Province-level proportion of IHD DALYs attributable to modifiable risk factors in China in 2016

The top two leading risk factors at the provincial level in 2016 showed homogeneity. High systolic blood pressure and high LDL cholesterol, which are metabolic risk factors, remained the two leading risk factors in all provinces, with the former being the leading risk factor in twenty-eight provinces and the latter in the remaining five provinces (Guangxi, Hainan, Hong Kong, Xinjiang, and Yunnan). The leading behavioral risk factor varied across provinces, with diet high in sodium being the leading behavioral risks in twenty-eight provinces and smoking in Gansu, Guangxi, Hainan, Tianjin, and Heilongjiang. For environmental risk factors, IHD DALYs attributable to ambient particulate matter pollution were highest in Tianjin and Beijing, and lowest in Tibet. Conversely, household air pollution from solid fuels accounted for the highest proportion of DALYs in Tibet (15.79%), and the lowest PAF was observed in Shanghai (0.44%), followed by Beijing (0.45%) and Tianjin (0.87%) (Fig. 3 and Additional file 1).

## Discussion

Most previous studies have only assessed the burden of IHD among overall populations without any further stratification regarding temporal or spatial patterns. To the best of our knowledge, this is the first trend analysis focusing on age-, sex-, and province-specific IHD burden over the years 1990 to 2016 including the periods 1990 to 2005 and 2005 to 2016. The results can provide important information for developing IHD prevention and control strategies.

### Age and sex disparities

The burden of IHD varied between ages, sexes, and provinces among the Chinese population. Our age-sex-specific analysis demonstrated that the IHD burden among seniors, particularly those aged 70+ years, was markedly higher than those under 49 years old, indicating that age was associated with the increased risk of IHD. Since this is related to a growing population and rapid aging, it is expected that the middle-aged and elderly individuals should be the foremost concern when managing the IHD burden in China [16, 17]. Marked discrepancies in the burden of IHD between sexes were found. YLDs in females were consistently higher than those in males whereas females experienced lower YLLs and DALYs. Behavioral patterns might partly explain the different trends seen in the IHD burden between sexes. In addition to males being more likely to have dreadful habits such as smoking, alcohol consumption, and poor diet which increases their baseline mortality rate of CVD [3], women tended to be active in seeking health care because they were in general more concerned about physical and mental health [10, 18]. These findings show that the IHD burden among middle-aged and elderly males should be more widely publicized to manage the overall IHD burden.

### Regional trends and disparities

Differences in the IHD burden existed between provinces of China. Most provinces saw a remarkable increase in IHD burden between 1990 and 2016. However, the age-standardised DALY rate experienced a sharp decline in several economically developed provinces, particularly in southeastern coastal areas such as Zhejiang, Hong Kong, and Macao. Socioeconomic status is one possible explanation for the provincial discrepancy, with poorer socioeconomic development being correlated with higher prevalence and death rates of diseases [11, 12, 19]. Equitable access to essential health services within provinces is a concern in China. The growing IHD burden over the past two decades is particularly obvious in some remote and less economically developed provinces, such as Guizhou and Yunnan, where there is generally lower public awareness of preventive self-care, limited education, weaker health services, and inability to pay for treatment. Furthermore, north-south and west-east disparities in medical resource allocation, environmental conditions, individual metabolism, distribution of risk factors, and lifestyles and behavioral habits also exacerbated the provincial discrepancy [8, 11, 13, 20, 21]. The regional disparities were observed across countries worldwide. High-income countries such as South Korea, Denmark, and Israel experienced a considerable reduction in age-standardised DALY rates during 1990–2016, whereas Bangladesh and Uzbekistan in lower-middle income regions had an opposite increasing trend [4]. Tailored strategies of mitigating the IHD burden should be priorities for these countries with limited economic development.

### Trends in IHD burden over time

Disparities in the IHD burden over time in China were observed. When comparing the variation in IHD burden in 1990–2005 and 2005–2016, we found that the rapid increase in YLLs had been effectively controlled in the most recent decade by the increase in YLDs. The progressive trend in the increase of YLD can be indicated by substantially increased prevalence of IHD. The China PEACE study showed national rates (per 100,000) of hospital admissions for ST-segment elevation myocardial infarction increased from 3.5 in 2001 to 15.4 in 2011. This rapidly rising incidence increased in its contribution to IHD prevalence [22]. Over the past two decades, improvements in health care services and medical treatments prolonged HALE of the Chinese population [13]. Unfortunately, the improvements contributed to the population growing and ageing, which increased the number of IHD cases, especially among the elderly aged > 65 years [4, 8, 20]. In addition, most old patients with IHD have comorbid peripheral atherosclerotic disease but guideline-recommend therapies are unavailable to them, which presents considerable challenges for disability control of IHD. While the IHD burden increased from 2005 to 2016, it did so at a relatively slower rate, with no significant differences in YLL in the elderly population during this period. Besides the increased number of older IHD patients, this can be partly explained by the enforcement of policies after 2005. National leadership has launched critical strategies and valuable prevention and control programs related to cardiovascular rehabilitation and tobacco control, along with widely advocating balanced diets and healthy lifestyles to ameliorate growing IHD hazards. In the new epoch seeking to achieve the “Healthy China 2030” [23] and the “13th Five-Year Public Health and Health Care Plan” [24], managing an increasing IHD burden is anticipated to be an uphill battle.

### Potential risk factors of IHD

Over the past two decades, demographic shifts, with transitions in economic systems, social structures, environmental factors, life styles, and medical treatments and health-care services have had a widespread and far-reaching impact on potential IHD risk factors [8, 25]. We found wide geographic variation in epidemiology of risk factors among provinces in 2016. Metabolic risks factors including high blood pressure and high LDL cholesterol were the leading contributors of IHD burdens, with diet high in sodium and smoking remaining the main sources of the IHD burden attributable to behavioral risks in China. Exposure to environmental risk factors was associated with economic development. Some provinces with weak economies, such as Tibet, had the highest IHD burden due to air pollution from solid fuels, with Beijing and Tianjin, rich in resources, experiencing the highest IHD burdens attributable to ambient particulate matter. Consistent with previous studies, our findings suggest the need to take tailored steps to mitigate exposure to risk factors such as physical inactivity, hypertension, high LDL-cholesterol levels, diabetes, and the low rates of education regarding the tertiary prevention of IHD [7, 26–31].

### Study strengths and implications for policies

Several studies paying attention to the increased burden of IHD in China have been carried out. Most previous studies mainly conducted at the local level with a limited time frame and failed to provide an overall temporal and geographical trend analysis considering both demographic characteristics and provincial disparities [9–11, 16, 17, 32]. Our study provided comprehensive estimates of the IHD burden among Chinese and subgroup populations according to age, sex, and regions, with an in-depth analysis of regional differences in risk factors at the provincial level. Through this we have uncovered some potentially valuable insights which can influence policy decisions. Firstly, we call for priorities aimed at improving the health level of general populations, particularly among middle-aged and elderly males engaging in high-risk behaviors. Better guidance in implementing targeted health policies at the provincial level needs to be emphasized, while also allowing for more accessible approaches based on different economic levels across provinces of China. Health authorities should pay more attention to the issue of “health inequality” and ensure that everyone is available to psychological well-being, regardless of their socio-economic status, gender, ethnicity, and residency. Our findings also uncover valuable insights for informing local governments of tailored strategies and programs to reduce the prevalence of IHD risk factors.

In summary, this study calls for delivering health care services which will improve the health status of the whole population, and for bolstering the economic and social advancements, particularly in the 70+ elderly, males, and residents in economically disadvantaged provinces of China, such as Guizhou and Yunnan.

### Study limitations

While it improves on previous studies, this study has some limitations. Although our analysis of IHD burden was conducted at national and provincial levels, the disease burden at the local level and urban-rural discrepancies were not examined. The gaps between assessments at national/provincial levels and district levels were somewhat limited to measure a shift in the local disease burden and effectively and specifically influence public policies, which underscores the need for future district level studies [33]. Although the GBD 2016 study has updated the epidemiological data and analytic methodology, changes in diagnostic technology over time may cause inevitable measurement errors in the acquisition of data [2–5]. With regard to the estimation of YLDs, the calculation of disability weighting is based on multinational results, and precise data on non-fatal outcomes of IHD are insufficient, which may cause uncertainty for China [2]. In addition, due to unavailability of clinical data, we cannot provide analyses of essential characteristics of patients and clinical variables, which should be worthy of our future studies.

## Conclusion

Our findings identified variations in the IHD burden among different ages, sexes, and provinces in China over the periods 1990 to 2016, 1990 to 2005, and 2005 to 2016. Some targeted public health strategies which take account subgroup populations’ characteristics in various regions are expected to be adopted, particularly in middle-aged and elderly males and those in economically disadvantaged provinces with resource constraints. We also found that premature death remained the leading cause of IHD burden in China along with the increased risk of disability. Although the increase of IHD burden has been effectively controlled after 2005, better guidance on measures at the provincial level which aim to protect vulnerable populations should be urgent priorities. The central and provincial health authorities also need to make concerted efforts to reduce the IHD burden inequality between economically rich and deprived regions. Considerable discrepancies were observed in IHD DALYs attributable to behavioral, environmental, and metabolic risk factors across provinces.

## Supplementary information

**Additional file 1.** Population-attributable fraction of IHD DALYs for modifiable risk factors in 33 provinces/regions of China in 2016.

## Data Availability

Data are available in the 2016 Global Burden of Disease Study.

## Abbreviations

IHD: Ischemic heart disease
NCDs: Non-communicable disease
WHO: World Health Organization
GBD: Global burden of disease
CVDs: Cardiovascular diseases
YLDs: Years lived with disability
YLLs: Years of life lost
DALYs: Disability-adjusted life years
ICD: International statistical classification of diseases
CDC: Center for Disease Control and Prevention
CRA: Comparative risk assessment
TMREL: Theoretical minimum risk exposure level
PAF: Population attributable fraction
