# Supplementary material for: Temporal and spatial trends of ischemic heart disease burden in Chinese and subgroup populations from 1990 to 2016: socio-economical data from the 2016 global burden of disease study
Source: BMC Cardiovasc Disord. 2020 May 24;20:243. doi: 10.1186/s12872-020-01530-0 (PMC7247238; doi:10.1186/s12872-020-01530-0)
Supplement: Supplementary file 1 — Additional file 1. Population-attributable fraction of IHD DALYs for modifiable risk factors in 33 provinces/regions of China in 2016. [file 12872_2020_1530_MOESM1_ESM.doc]

|  | a | b | c | d | e | f | g | h | i | j | k | l | m | n | o | p | q | r | s | t | u | v | w | x |
| --- | --- | --- | --- | --- | --- | --- | --- | --- | --- | --- | --- | --- | --- | --- | --- | --- | --- | --- | --- | --- | --- | --- | --- | --- |
| Anhui | 52.68 | 43.53 | 37.45 | 14.53 | 27.76 | 22.40 | 25.03 | 18.80 | 12.18 | 12.62 | 13.38 | 13.31 | 10.02 | 11.73 | 9.34 | 8.34 | 6.73 | 6.02 | 5.04 | 5.03 | 1.57 | 1.00 | 0.56 | 0.21 |
| Beijing | 52.24 | 46.37 | 32.50 | 16.81 | 28.04 | 21.00 | 21.39 | 15.24 | 4.91 | 22.78 | 19.15 | 9.63 | 7.64 | 10.33 | 9.31 | 7.94 | 5.41 | 0.45 | 3.92 | 3.66 | 4.41 | 0.98 | 2.06 | 0.82 |
| Chongqing | 54.17 | 42.63 | 34.89 | 13.59 | 24.39 | 20.94 | 23.37 | 17.21 | 10.08 | 12.06 | 12.89 | 11.72 | 9.04 | 10.38 | 8.54 | 8.28 | 6.14 | 5.14 | 8.26 | 4.85 | 2.20 | 0.94 | 0.67 | 0.27 |
| Fujian | 50.09 | 47.98 | 37.22 | 15.45 | 27.60 | 21.67 | 23.49 | 17.03 | 7.56 | 13.38 | 12.40 | 11.17 | 8.67 | 10.97 | 9.37 | 8.12 | 6.76 | 3.14 | 8.35 | 4.90 | 1.25 | 1.00 | 1.30 | 0.51 |
| Gansu | 53.98 | 43.72 | 26.85 | 13.14 | 30.72 | 23.65 | 26.36 | 20.64 | 13.75 | 13.80 | 10.72 | 14.51 | 10.73 | 12.58 | 9.78 | 7.71 | 7.58 | 9.96 | 9.53 | 4.96 | 2.67 | 1.05 | 0.49 | 0.18 |
| Guangdong | 50.88 | 48.30 | 32.59 | 15.96 | 28.54 | 22.15 | 23.67 | 17.18 | 6.91 | 12.84 | 14.45 | 11.19 | 8.65 | 11.30 | 9.74 | 8.28 | 6.22 | 2.23 | 10.12 | 5.61 | 3.49 | 1.04 | 1.51 | 0.60 |
| Guangxi | 47.55 | 48.95 | 23.06 | 13.41 | 28.45 | 3.42 | 26.10 | 20.08 | 12.24 | 12.31 | 12.30 | 13.91 | 10.31 | 12.42 | 9.87 | 7.16 | 7.09 | 6.97 | 8.00 | 4.97 | 2.49 | 1.05 | 0.68 | 0.25 |
| Guizhou | 55.87 | 47.87 | 36.52 | 12.65 | 32.40 | 23.83 | 26.46 | 20.71 | 14.65 | 11.56 | 9.85 | 14.87 | 10.93 | 12.78 | 9.77 | 5.73 | 6.48 | 9.90 | 10.24 | 5.08 | 0.72 | 1.05 | 0.38 | 0.15 |
| Hainan | 47.02 | 50.05 | 28.29 | 17.22 | 28.81 | 23.23 | 25.80 | 19.40 | 11.47 | 11.13 | 8.60 | 13.54 | 10.14 | 12.23 | 9.82 | 7.72 | 6.54 | 7.18 | 7.77 | 4.89 | 2.36 | 1.04 | 0.79 | 0.29 |
| Hebei | 57.56 | 48.82 | 40.10 | 15.96 | 30.07 | 23.70 | 26.17 | 20.11 | 10.28 | 21.99 | 18.83 | 13.24 | 10.05 | 12.44 | 10.17 | 8.01 | 7.11 | 3.84 | 7.18 | 7.45 | 3.31 | 1.09 | 1.02 | 0.38 |
| Heilongjiang | 57.96 | 48.85 | 30.78 | 16.77 | 34.38 | 23.51 | 26.10 | 20.26 | 11.18 | 19.25 | 13.86 | 13.47 | 10.05 | 12.36 | 9.99 | 6.48 | 7.49 | 5.21 | 5.57 | 5.27 | 2.16 | 1.08 | 0.81 | 0.30 |
| Henan | 54.83 | 44.56 | 38.61 | 14.49 | 26.14 | 22.99 | 25.60 | 19.64 | 11.12 | 20.97 | 16.51 | 13.22 | 9.95 | 11.97 | 9.67 | 7.25 | 6.67 | 5.24 | 10.72 | 4.96 | 2.36 | 1.05 | 0.77 | 0.29 |
| Hubei | 46.64 | 50.42 | 37.58 | 14.28 | 21.12 | 20.61 | 13.44 | 9.68 | 9.29 | 16.48 | 11.57 | 9.10 | 3.87 | 11.26 | 9.12 | 8.36 | 5.77 | 2.80 | 2.08 | 4.99 | 5.04 | 0.98 | 2.64 | 2.09 |
| Hunan | 54.52 | 43.45 | 33.13 | 13.17 | 26.20 | 22.10 | 24.64 | 18.62 | 10.75 | 14.18 | 15.10 | 12.58 | 9.57 | 11.20 | 9.12 | 7.15 | 6.74 | 4.36 | 6.71 | 5.02 | 1.32 | 1.00 | 0.71 | 0.28 |
| Inner Mongolia | 53.76 | 45.01 | 36.41 | 14.07 | 28.12 | 22.50 | 25.14 | 19.36 | 11.35 | 12.30 | 13.80 | 13.05 | 9.86 | 11.56 | 9.28 | 9.02 | 6.90 | 5.59 | 9.83 | 5.39 | 3.23 | 1.01 | 0.67 | 0.26 |
| Jiangsu | 57.93 | 50.08 | 43.96 | 15.25 | 32.86 | 23.30 | 25.67 | 19.61 | 9.53 | 20.68 | 12.39 | 12.77 | 9.71 | 12.19 | 10.06 | 8.19 | 6.90 | 6.96 | 4.69 | 5.20 | 2.53 | 1.08 | 1.10 | 0.41 |
| Jiangxi | 53.80 | 42.97 | 35.76 | 15.14 | 26.08 | 21.05 | 22.50 | 15.21 | 6.74 | 16.58 | 15.74 | 10.43 | 6.94 | 9.61 | 9.08 | 7.93 | 5.79 | 2.35 | 10.57 | 5.22 | 1.93 | 0.99 | 1.34 | 0.54 |
| Jilin | 51.18 | 43.67 | 37.32 | 13.11 | 28.84 | 22.79 | 25.46 | 19.50 | 12.34 | 11.33 | 11.29 | 13.61 | 10.17 | 11.88 | 9.42 | 8.14 | 6.53 | 7.83 | 4.48 | 4.96 | 0.82 | 1.01 | 0.58 | 0.22 |
| Liaoning | 55.80 | 49.71 | 34.44 | 16.41 | 29.44 | 23.25 | 25.78 | 19.74 | 10.62 | 18.20 | 14.81 | 13.11 | 9.92 | 12.22 | 9.97 | 8.59 | 7.72 | 4.73 | 9.75 | 5.34 | 2.22 | 1.07 | 0.90 | 0.33 |
| Ningxia | 61.81 | 46.23 | 34.85 | 16.65 | 29.26 | 22.32 | 24.51 | 18.55 | 8.71 | 17.84 | 16.11 | 11.87 | 9.13 | 11.33 | 9.50 | 7.90 | 6.77 | 2.98 | 5.37 | 4.92 | 2.74 | 1.03 | 1.10 | 0.43 |
| Qinghai | 56.30 | 50.08 | 43.12 | 18.48 | 30.44 | 20.19 | 18.31 | 12.58 | 12.41 | 22.47 | 12.24 | 10.62 | 8.01 | 12.66 | 5.91 | 8.38 | 5.16 | 3.12 | 1.64 | 5.08 | 0.86 | 0.99 | 5.31 | 2.07 |
| Shaanxi | 53.75 | 46.08 | 33.55 | 12.83 | 32.69 | 24.11 | 26.76 | 20.67 | 12.16 | 16.75 | 14.02 | 14.25 | 10.50 | 12.98 | 10.33 | 7.60 | 7.42 | 6.63 | 6.41 | 4.97 | -0.09 | 1.09 | 0.75 | 0.28 |
| Shandong | 53.46 | 46.54 | 40.91 | 13.04 | 29.18 | 24.60 | 27.26 | 21.22 | 12.85 | 15.64 | 12.70 | 14.63 | 10.82 | 13.50 | 10.65 | 7.30 | 8.78 | 8.37 | 8.24 | 5.22 | 0.91 | 1.15 | 0.72 | 0.25 |
| Shanghai | 53.60 | 44.57 | 39.87 | 12.66 | 29.31 | 23.20 | 25.83 | 19.82 | 11.70 | 14.77 | 14.47 | 13.54 | 10.14 | 12.19 | 9.79 | 7.84 | 6.72 | 6.10 | 9.65 | 4.95 | 2.44 | 1.05 | 0.73 | 0.27 |
| Shanxi | 56.16 | 45.64 | 40.45 | 15.18 | 28.65 | 21.47 | 23.42 | 17.40 | 7.69 | 18.18 | 16.40 | 11.07 | 8.62 | 10.79 | 9.20 | 9.05 | 6.29 | 3.60 | 3.44 | 4.51 | 2.36 | 1.00 | 1.19 | 0.47 |
| Sichuan | 51.55 | 46.64 | 30.42 | 16.09 | 20.37 | 19.80 | 20.42 | 13.43 | 5.41 | 15.72 | 14.68 | 9.12 | 7.51 | 9.84 | 8.82 | 12.81 | 4.10 | 0.44 | 4.89 | 4.95 | 3.30 | 0.93 | 1.59 | 0.64 |
| Tianjin | 56.17 | 45.51 | 37.91 | 15.79 | 30.51 | 24.05 | 26.66 | 20.32 | 11.79 | 18.61 | 15.42 | 14.04 | 10.47 | 12.98 | 10.40 | 7.88 | 6.09 | 5.69 | 6.77 | 5.33 | 3.26 | 1.10 | 0.81 | 0.30 |
| Tibet | 53.51 | 45.58 | 35.27 | 13.54 | 27.77 | 22.38 | 24.96 | 18.98 | 11.57 | 13.24 | 12.86 | 13.09 | 9.82 | 11.50 | 9.25 | 7.94 | 6.76 | 6.35 | 10.41 | 4.50 | 1.49 | 1.01 | 0.62 | 0.24 |
| Xinjiang | 53.33 | 47.36 | 27.09 | 18.48 | 29.49 | 21.98 | 20.08 | 17.16 | 4.97 | 21.39 | 20.08 | 10.50 | 8.35 | 10.95 | 10.50 | 8.35 | 5.70 | 0.87 | 4.59 | 5.09 | 4.32 | 1.02 | 1.85 | 0.71 |
| Yunnan | 58.93 | 48.43 | 45.50 | 11.58 | 29.37 | 26.06 | 28.83 | 22.67 | 14.79 | 12.03 | 5.92 | 16.05 | 11.84 | 14.60 | 11.24 | 7.49 | 8.82 | 15.79 | 12.00 | 5.16 | -1.52 | 1.21 | 0.67 | 0.22 |
| Zhejiang | 51.53 | 51.68 | 50.44 | 18.47 | 28.68 | 25.66 | 28.31 | 22.01 | 11.99 | 24.95 | 16.69 | 14.91 | 11.03 | 14.25 | 11.41 | 7.56 | 6.81 | 6.54 | 6.37 | 5.53 | 3.61 | 1.20 | 1.03 | 0.36 |
| Hong Kong Special Administrative Region of China | 48.16 | 50.56 | 36.27 | 13.02 | 31.53 | 24.56 | 27.25 | 21.13 | 13.38 | 11.53 | 9.39 | 14.86 | 10.97 | 13.42 | 10.47 | 7.91 | 7.78 | 9.88 | 8.42 | 5.04 | 1.44 | 1.11 | 0.64 | 0.23 |
| Macao Special Administrative Region of China | 53.50 | 45.59 | 35.58 | 14.56 | 28.04 | 21.21 | 22.30 | 15.46 | 7.20 | 13.26 | 14.78 | 11.12 | 8.13 | 10.64 | 9.38 | 9.39 | 5.31 | 1.73 | 7.08 | 5.08 | 1.47 | 0.99 | 1.63 | 0.64 |

**e Table. Population-attributable fraction of IHD DALYs for modifiable risk factors in 33 provinces/regions of China in 2016**

*IHD: ischemic heart disease;

DALY: disability-adjusted years;

a: High systolic blood pressure; b: High LDL cholesterol; c: Diet high in sodium; d: High fasting plasma glucose; e: Smoking; f: Diet low in whole grains; g: Diet low in nuts and seeds; h: Diet low in seafood omega-3 fatty acids; i: Diet low in vegetables; g: High body-mass index; k: Ambient particulate matter pollution; l: Diet low in fruits; m: Diet low in polyunsaturated fatty acids; n: Diet low in fiber; o: Diet low in legumes; p: Low physical activity; q: Secondhand smoke; r: Household air pollution from solid fuels; s: Lead exposure; t: Impaired kidney function; u: Alcohol use; v: Diet high in trans fatty acids; w: Diet high in sugar-sweetened beverages; x: Diet high in processed meat
